# Supply chain viability: conceptualization, measurement, and nomological validation

**DOI:** 10.1007/s10479-021-03974-9

**Published:** 2021-03-08

**Authors:** Salomée Ruel, Jamal El Baz, Dmitry Ivanov, Ajay Das

**Affiliations:** 1 MOSI department - CSR excellence center, KEDGE Business School, Domaine de Luminy, Rue Antoine Bourdelle, 13009 Marseille, France; 2 Ibn Zohr University Agadir, ERETTLOG, Agadir, Morocco; 3 Department of Business and Economics, Berlin School of Economics and Law, 10825 Berlin, Germany; 4 Operations Management Group, N.P. Loomba Dept. of Management, Zicklin School of Business, Baruch College, CUNY, One Bernard Baruch Way, New York, NY 10010 USA

**Keywords:** Supply chain viability, COVID-19 pandemic, Measurement, Scale development, Second-order construct

## Abstract

Supply chain viability (SCV) is an emerging concept of growing importance in operations management. This paper aims to conceptualize, develop, and validate a measurement scale for SCV. SCV is first defined and operationalized as a construct, followed by content validation and item measure development. Data have been collected through three independent samplings comprising a total of 558 respondents. Both exploratory and confirmatory factor analyses are used in a step-wise manner for scale development. Reliability and validity are evaluated. A nomological model is theorized and tested to evaluate nomological validity. For the first time, our study frames SCV as a novel and distinct construct. The findings show that SCV is a hierarchical and multidimensional construct, reflected in organizational structures, organizational resources, dynamic design capabilities, and operational aspects. The findings reveal that a central characteristic of SCV is the dynamic reconfiguration of SC structures in an adaptive manner to ensure survival in the long-term perspective. This research conceptualizes and provides specific, validated dimensions and item measures for SCV. Practitioner directed guidance and suggestions are offered for improving SCV during the COVID-19 pandemic and future severe disruptions.

## Introduction

Paraphrasing Charles Darwin’s central thesis, “It is not the most intellectual of the species that survives; it is not the strongest that survives; but the species that survives is the one that is able best to adapt and adjust to the changing environment in which it finds itself” (Megginson 1963)*.* Yet Darwin envisaged change and adaptation over generations and eons—how would sudden catastrophic changes affect organisms? How would they adapt to ensure the immediate need for survival?

In the context of supply chains (SC), the COVID-19 crisis has rendered sudden and catastrophic change in the business environment and beyond, impacting and disrupting operations and SCs in terms of scale, complexity, severity, and duration of impact. With the COVID-19 pandemic, some novel context has been unveiled which goes beyond an instantaneous event-driven understanding of disruptions and can be described as an *SC crisis* characterized by long and severe uncertainty of current and future conditions and entailing extensions toward SC viability. To survive or maintain a viable SC can be a challenge when faced with such ‘super disruptions’ that can radically change operational conditions over long durations (Ivanov 2020a; Singh et al. 2020; ElBaz and Ruel 2021; Jang et al. 2021). Despite remarkable progress in our understanding of the disruptions, the COVID-19 pandemic has revealed research gaps with regard to such super-disruptions. We posit that survival and adaptation in confronting such super-disruptive changes require a special property—the capability to survive, to remain viable—that is, SC Viability (SCV) (Ivanov 2021c).

Supply chain viability (SCV) is an emerging concept of growing importance in operations management in times of COVID-19 pandemic and well recognized by practitioners (Ivanov 2020b). In recent decades, an increasing number of uncertainties and disruptions has stimulated researcher interest in the theme of SC resilience, a firm’s capability to recover from disruptions to meet customer demand, ensure target performance, and maintain operations in vulnerable environments (Dubey et al. 2019; Hosseini et al. 2019; Sawik 2020; Choi 2020; Azadegan and Dooley 2021). SC resilience has been viewed as the ability to bounce-back and recover towards an “old normal” (Fiksel 2006; Pettit et al. 2010) once disrupted. The COVID-19 pandemic is a very special kind of disruption (Gunessee and Subramanian 2020; Ivanov 2020a, b; Paul and Chowdhury 2020; Queiroz et al. 2020; Ivanov 2021b; Tang et al. 2021), and it has raised novel questions within a decision-making context which frequently go beyond the scope of SC resilience (Hosseini et al. 2020; Dolgui and Ivanov 2021; Ivanov 2021a).

The COVID-19 pandemic has posed a new disruption context for firms, stimulating attempts to define new theoretical lens which overarch existing resilience capabilities (Chowdhury and Quaddus 2017). Craighead et al. (2020) use ten different theories—resource dependence theory, institutional theory, game theory, and others—to draw out research questions, offering ways for simultaneous transformation and resilience, i.e. transiliency (i.e., the ability to simultaneously restore some processes and change—often radically—others). Hosseini et al. (2020) elaborate on the open-system view and propose novel metrics to measure resilience in the open-system context.

Wieland (2020) proposed a panarchy framework for SCs based on adaptive cycles linked across SC, political-economical, and planetary levels on scales of time, space, and meaning. Considering SC structures and processes to be reconfigurable (i.e., “fluid”), Wieland reinterprets the SC as a social–ecological system replacing a static view of SC management with a vision of “dancing the SC” which is in line with the structural dynamics control approach by Ivanov et al. (2010), reconfigurable SC framework by Dolgui et al. (2020), and the viable SC framework by Ivanov (2020b).

In the emerging spirit of re-thinking and re-inventing SC management driven by the pandemic context, we posit that certain aspects of this pandemic-related context can be approached using the notion of SCV. In situations where SCs were literally crumbling, the question no longer concerned bouncing back and recovering to some “normal” state, but rather *how* to adapt and survive in radically changed internal and external conditions. To address this and related questions, we build on a novel theoretical underpinning of SCV (Ivanov 2020b).

“Viable Supply Chain (VSC) is a dynamically adaptable and structurally changeable value-adding network able to (i) react agilely to positive changes, (ii) be resilient to absorb negative events and recover after the disruptions, and (iii) survive at the times of long-term, global disruptions by adjusting capacities utilizations and their allocations to demands in response to internal and external changes in line with the sustainable developments to secure the provision of society and markets with goods and services in long-term perspective (Ivanov 2020b).”

**Fig. 1:**
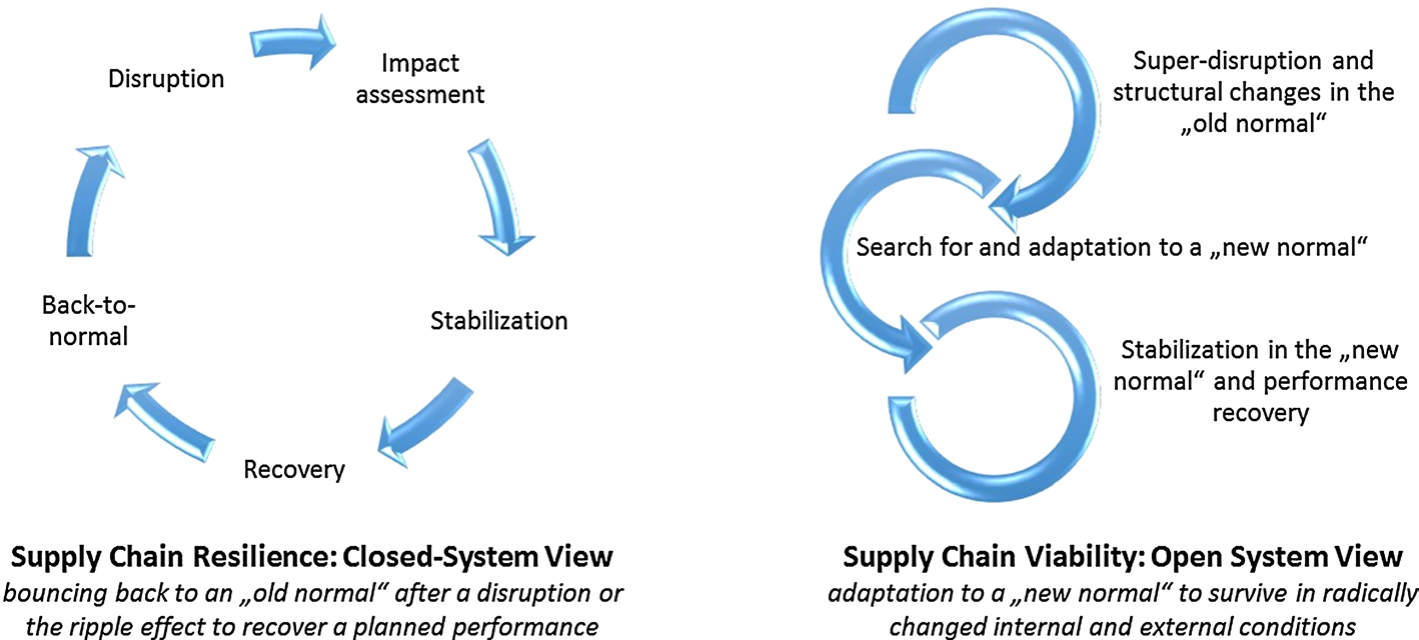
SC viability as an extended resilience perspective

SCV can be viewed from an overarching adaptation perspective that extends the SC resilience notion of a closed-system, “bounce-back” view, with a viable, open SC system perspective incorporating “bounce-forward-and-adapt” options (see Fig. 1).^1^

SCV is an emerging but increasingly recognized capability in industry, with practitioners valuing and using it as a critical resource in COVID-19 stricken business environments (Hofmann and Langner 2020). Examples abound in industry. With demand plummeting, companies such as LVMH (perfumes) and Skyrora (rockets) adapted to manufacturing hand sanitizer. The SC for sanitizers is very different from that for perfumes or rockets. Yet, these companies made the adaptation in record time, making rapid structural changes to their existing SCs and other areas. Similarly, faced with a sales decline of 90% in the China market, automotive manufacturer BYD Co. switched to making surgical masks, while Ford and GM quickly adapted to making ventilators, all at extremely short notice (Wade and Bjerkan 2020). However, an empirical examination of SCV is still missing in the literature. One reason for the lag between practice and theory could potentially be the lack of a validated scale for SCV.

The purpose of the study is to theorize, operationalize, and develop an empirical measurement scale for SCV. A review of the extant literature reveals that a theoretically grounded, comprehensive conceptualization and measurement of SCV is lacking. Our study is the first to address this deficiency by undertaking an empirically driven study to develop and validate a hierarchical and multidimensional measurement scale for SCV. Churchill’s (1979, 1995) protocol is undertaken to develop a hierarchical and multidimensional measurement scale for SCV. We define and operationalize SCV, with subsequent content validation and reliability assessment. Scale psychometric properties are established, with convergent, discriminant, and nomological validity examinations using multiple samples.

Our conceptualization, development, and nomological validation of a measurement scale for SCV make several, substantive contributions. *First*, our study enables the identification of methodical commonalities and differentiations of viability and resilience in order to frame SCV as a specific construct. Considering the nascent and rarely defined nature of the SCV concept, there appears an urgent need to clearly identify the dimensions of this complex construct. *Second*, our study represents a pioneering research initiative, providing the research community with the first empirically derived and validated scale for examining SCV as a distinct construct. *Third*, our findings reveal adaptability as the central perspective of SCV contributing to building the theory of SC management during a pandemic. The major concept of the viable SC (Ivanov 2020b) —adaptability as an ability to redesign the SC in the face of severe changes in its environment by relying on feedback mechanisms—is confirmed and extended. Our findings confirm that SCV is a hierarchical and multidimensional construct, which is reflected by organizational structures and resources and dynamic design capabilities. Finally, our research provides guidance to practitioners on SCV at a granular practice level, and confers on ways to improve SCV in COVID-19 pandemic times and future severe disruptions.

The rest of the paper is structured as follows. Section 2 presents a review of closely related literature. Section 3 describes the scale development protocol and methodology, including scale psychometric properties. Section 4 discusses the results and associated implications. Section 5 concludes the research with a discussion of study contributions and limitations, and a brief deliberation on opportunities for future research.

## Literature review

### Defining and distinguishing supply chain viability

The scale development process begins with construct definition and boundary delineation. We briefly trace the origins of systems viability and then transit to a discussion on SCV. The concept of system viability was first developed in ecology and biological systems (Aubin 1991) and cybernetics. The Viable System Model by Beer (1981) and ecology modelling perspectives from Aubin (1991) are inspirations for the emerging concept of SCV: they highlight the ability of a system to survive in a turbulent environment. By analogy, Ivanov and Dolgui (2020b) point out that SCs can be compared to the complex nature systems.

Ivanov (2020b) sees SCV as “the ability to maintain itself and survive in a changing environment over a long period of time through a redesign of the structures and replanning of economic performance with long-term impacts.” He defines three major pillars of SCV, i.e., the viable SC model, a multi-structural view of SC viability, and an ecosystem of a viable SC. The viable SC model is based on the development of multiple, alternative structural network designs for supply–demand allocations during normal, disruptive and super disruptive times, and importantly, the establishment and control of adaptive mechanisms for transitions between these structural designs. The multi-structural view decomposes viable SC into organizational, informational, process-functional, technological, and financial structures and resources, spanning various management and organizational perspectives. The ecosystem view of the viable SC entails major feedback cycles in SC network interactions with environment.

A SCV based SC design would have the potential to rapidly serve new markets, and/or pivot to new SCs for new products for business survival imperatives. The Panera Bread chain, having lost about 50% of its largely indoors business to COVID-19, adapted to a new SC in order to offer staple groceries along the traditional soups and bread. Burger chain Fuddruckers sold toilet paper, gloves, and bleach at specific locations—products far removed from its regular fast food product line, requiring entirely different SC infrastructure (Taylor 2020). A firm with SCV capability could also tap non-traditional supply markets for its existing products, in order to meet disruption induced surges in demand, as well as compensate for sudden deficiencies in its regular SCs. Amazon turned to demand decline hit Lyft for warehouse and logistical staffing needs, with the latter directing its employees to Amazon positions (Statt 2020).

Gathering the above discussion, SCV offers a means for the long-term maintenance of survivability under different and ever-changing environmental conditions. We operationalize SCV through its four primary dimensions of “Organizational structures and resources”, “Dynamic design capabilities”, “Time window”, and “Operational performance” following the SCV framework proposed in (Ivanov 2020b).

### Supply chain viability dimensions

**Fig. 2:**
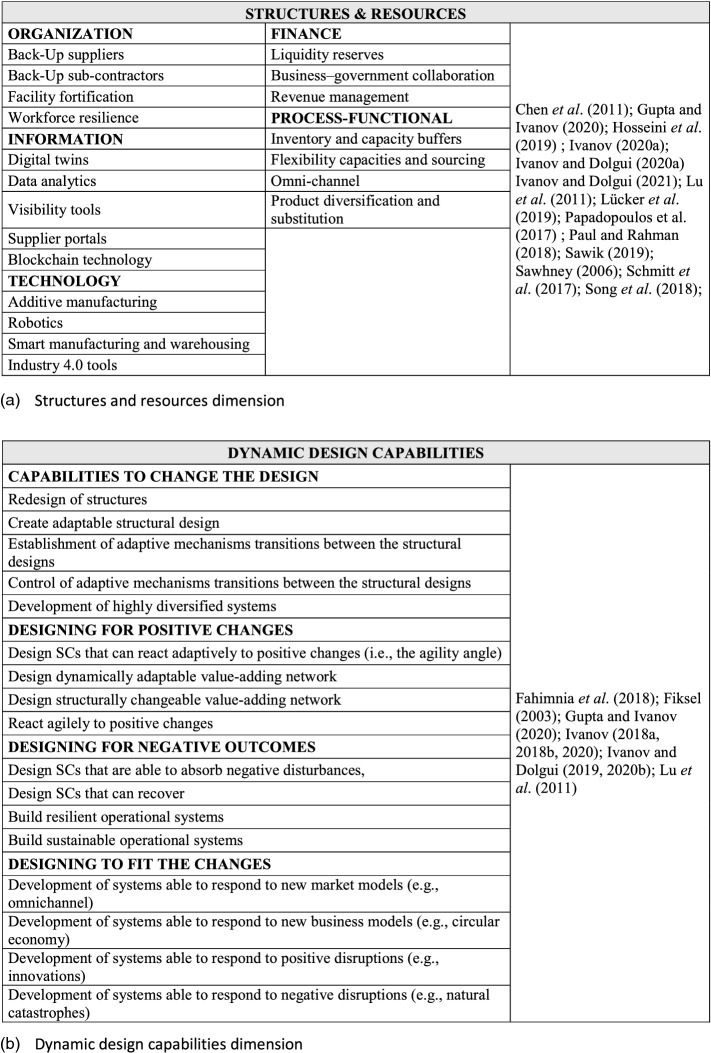

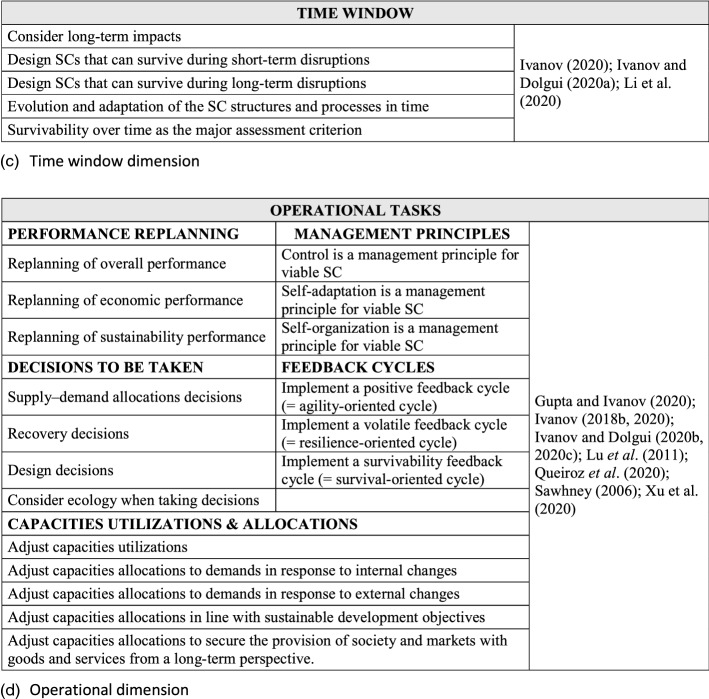
Supply chain viability dimensions

In Fig. 2, we summarize major SCV dimensions as posed in Ivanov (2020b). We use a triangulation of the SCV notion from (Ivanov 2020b) which is comprised of a multi-structural viability view, Viable SC Model (Tables 1 and 2), and SC ecosystem view (Fig. 2). In their totality, these complementary parts of the SCV concept are comprehensively addressed in our study when developing and validating the SCV measurement scale.

**Table 1:** Demographic profile of field study respondents

N	Length	Job position	Industry	Experience in SCM (years)
I1	1h 07 min	Supply Chain Manager (freelance)	Consulting	28
I2	46 min	Global distribution and Supply Chain project manager	Pharmaceuticals	16
I3	59 min	Supply Chain Manager	Telecom	23
I4	51 min	Supply Chain Manager	Aeronautics	14
I5	49 min	Supply Chain Consultant	Consulting (in agribusiness)	24
I6	1h 08	Supply Chain Director	Agrofood	32
I7	52 min	VP supply chain	Beauty & care	25
I8	58 min	Supply Chain Director freelance	Consulting (trading)	28
I9	1h13min	Supply Chain Manager (freelance)	Consulting (all)	32
I10	55 min	Supply Chain Manager	Automobile	14
I11	50 min	Supply Chain Consultant	Consulting (all)	14
Summary	57 min on average	All are at management or top management positions	Several types of industries	23.7 years on average

**Table 2:**
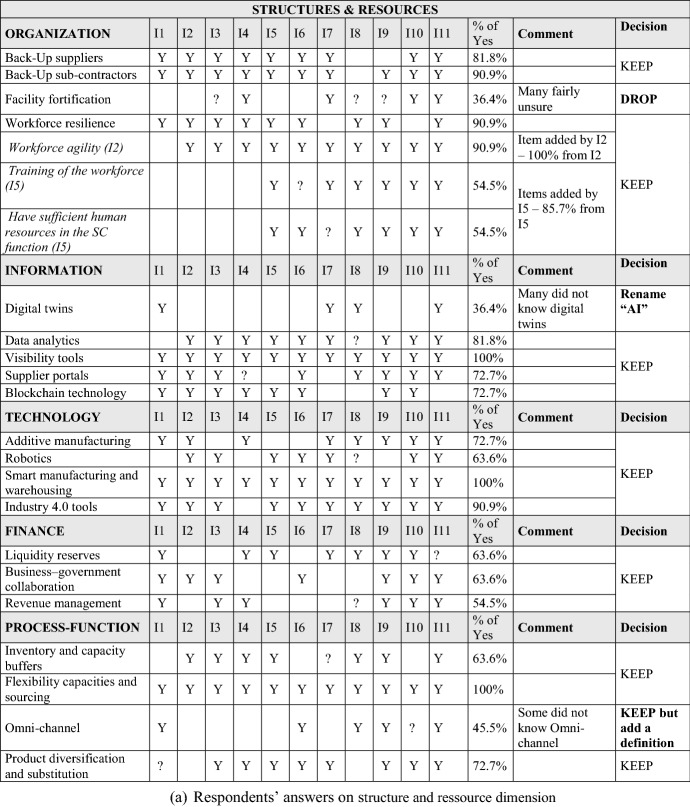

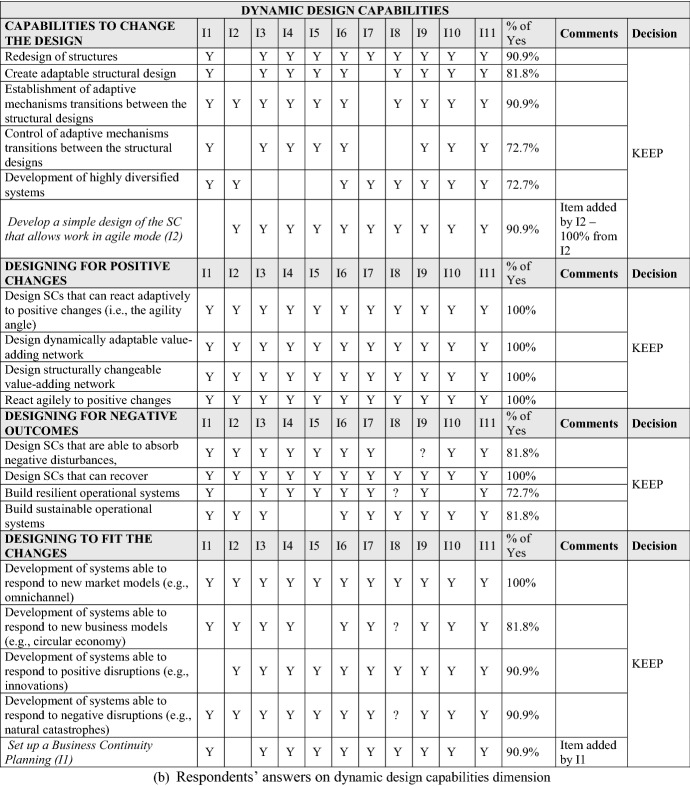

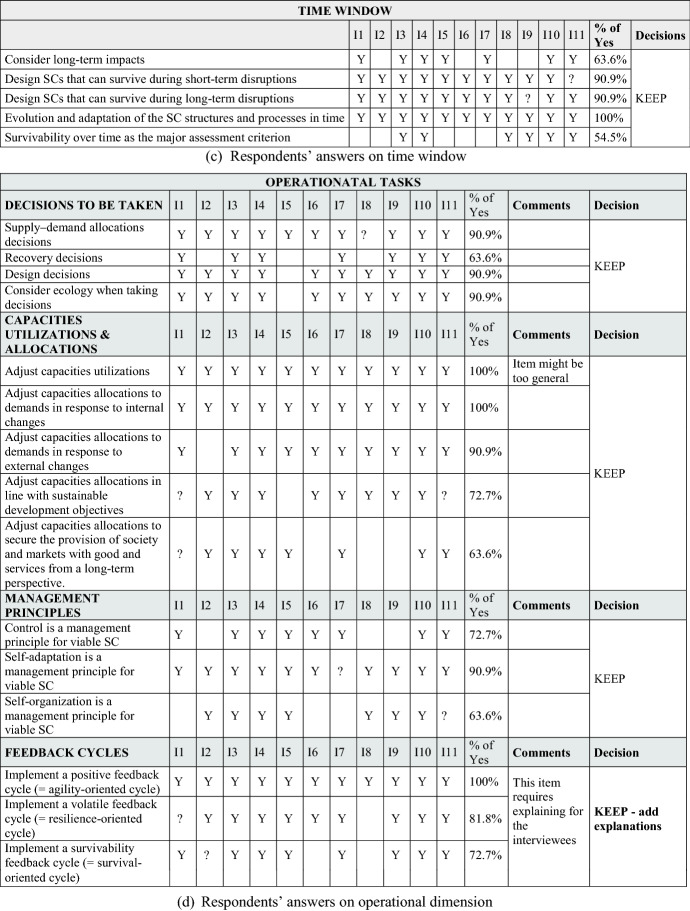
Respondents’ answers on SCV dimensions

#### Organizational structures and resources for SCV

Building on Ivanov’s (2020b) multi-structural view of SCV, Fig. 2 offers a systemic view of organizational structure and resources for SCV based on the literature around the stability, robustness, resilience, and viability concepts. Ivanov and Dolgui (2020b) propose that the SCV concept is embedded in multiple structures and associated resources.

#### Dynamic design capabilities for SCV

Another important aspect of SCV are dynamic design capabilities that facilitate fast transitions among (and testing of) alternative multiple structural SC designs, to meet volatile supply and demand conditions (Teece et al. 1997; Eisenhardt and Martin 2000; Winter 2003; Caniato et al. 2013). Ivanov (2020b) underlines the need for established and manageable adaptive mechanisms owing to the fact that it is nearly impossible to predict all possible future disruptions, and a-priori match respective SC designs to emergent scenarios. Quick plug and play adaptive mechanisms such as dynamic design capabilities are useful. Figure 2 shows the factors and items included in dynamic design capabilities.

#### Time window aspect of SCV

As viability has the objective of both helping SCs to meet sustainability objectives (Queiroz et al. 2020) and survive in time over disruptions, the time window is long-term. Several ideas around “time” can be found in the literature, as presented in Fig. 2. In the context of SCV, Ivanov and Dolgui (2020b) specified that “long-term” means “no fixed time window” when analyzing the SC.

#### Operational performance aspect of SCV

The literature also discusses operational tasks that, when undertaken, could improve SCV. Figure 2 synthesizes these aspects using the Ivanov’s (2020b) framework of SC ecosystem.

## Methodology

We develop the SCV scale in four phases. Phase 1 operationalizes SCV using previous conceptualization and cited literature. Phase 2 engages with the instrument development process. We use in-depth interviews and discussions with functional experts to sort item measures, possibly find new dimensions/variables, strengthen literature based content validity, and establish face validity. Phase 3 gathers data from a survey sample for examining scale psychometric properties. Fresh data is collected from a second, separate sample to confirm scale psychometric properties. Phase 4 theorizes and conducts a test of nomological validity, using data from a third, new sample. We find consistency in factor structures and construct validity. The sequence in our scale development is generally patterned on Churchill’s (1979) and Hensley’s (1999) paradigms.

### Phase 1: operationalization of supply chain viability

To operationalize the dimensions of SCV, this study began by investigating the commonly cited items for each dimension of SCV, as outlined in the literature review section. The literature review reveals that SCV is perceived as a multidimensional and hierarchical concept. We identified four primary dimensions that reflect SCV: Organizational structures and resources, Dynamic design capabilities, Time window, and Operational aspects. The literature review also revealed multiple sub-dimensions for these primary dimensions. The SCV concept being emergent (Ivanov 2018b, 2020b), an exploratory qualitative discussion with subject matter experts was undertaken to confirm the four dimensions of SCV and its sub-dimensions, and conceivably find additional dimensions and factors.

### Phase 2: instrument development process

The formal instrument development process began with a qualitative field study based on the cited literature. Both item creation and item sorting were undertaken for scale development. More precisely, we sought content validity by enumerating SCV primary dimensions and sub-dimensions, identifying and selecting a pool of items for each SCV sub-dimension (see Fig. 2). Since SCV is as yet, an embryonic concept, our initial pick of sub-dimensions and item measures was necessarily broad, subsequently re-examined and refined with input from expert SC professional perspectives.

We obtained qualitative data from 11 in-depth interviews conducted in June 2020 with SC professionals in France from diverse industrial and consulting companies. Table 1 presents the respondent profile.

In each case, we asked interviewees to answer similar questions asking about their views of SCV and its dimensions. All interviews were recorded and scripted for codification and analysis purposes. A thematic content analysis of verbatim using NVIVO12 software was conducted by one researcher from our team: the interviews were coded based on dimensions found in the literature, and this researcher remained open to the possibility of discovering other dimensions and factors. To verify the validity of this coding, a double coding procedure was conducted by a second researcher from our team on a third of the transcript with the list of thematic codes. This procedure showed a high agreement between both researchers and thus the validity of the original coding is ensured (Huberman and Miles 1994).

The interviews surfaced additional potential factors and led us to dismiss others, improving the content and face validity of the measurement instrument. For the sake of brevity, we do not present the details of the qualitative study here. Table 2 summarizes the factors and variables derived from the interviews and literature review.

First, Table 2 presents answers from the 11 SC professionals to the question “Does […] contribute to SCV?” The initial dimensions were literature based, with others added later, grounded in respondent suggestions. We also asked an open-question about organizational structures and resources for SCV. Second, Table 2 describes answers from the respondents regarding dynamic design capabilities. An open ended question suggested three additional factors beyond our initial literature based factor list: Redesigning brings more visibility, Redesigning should consider knowledge management, and Redesigning can help to bypass problems in the SC. We incorporated these in our analysis. Third, Table 2 shows respondent perspectives on literature grounded factors regarding the ‘time window’ aspect of SCV. Our open ended question did not raise any new factors for consideration. Finally, Table 2 summarizes the 11 interviewees’ answers to specific operational tasks that appeared in the cited literature. Additional factors emerged from our interviews. These supplemented our initial list of factors and items for this sub-dimension. Summing up, the interviews influenced both factor (and item) generation and deduction. The resulting factors and items provided a comprehensive representation of the SCV construct.

### Phase 3: psychometric evaluation

In line with best paradigms in scale development Churchill (1979, 1995), we tested the measurement scale over two independent samples: a control sample of 163 respondents then the final sample of 265 respondents. To address the possibility of a non-response bias, we incorporated the standard testing of early and late responders to identify the presence of structural differences. Chi-square tests between early and late responders were conducted across all relevant groups that describe the sampling frame (size, sector, and experience). The results of these analyses indicate that there does not appear to be a significant issue with any biases associated with non-response.

The control and final samples contained in excess of 90% and 92% respondents with SC, purchasing and operations expertise, respectively—lending assurance to the subject matter quality of the responses. About 93% and 84% of respondents from the control and final samples, respectively, had between 5 years to 20 or more years of job experience, providing added credibility to the quality of the information provided. The data came from a mix of small to large sized firms drawn from a variety of industry sectors, which fact bolsters the external validity of our findings.

#### Exploratory factor analysis

The aim of EFA is to assess the unidimensionality and structure of constructs (Netemeyer et al. 2003). In general, EFA can be conducted through “within-block”, i.e., each construct in isolation, or “across-block”, i.e., all items across constructs (Ponsignon et al. 2020). We adopted both approaches in this paper, because a set of variables that were identified as unidimensional by the existence of a single factor within the block might fail to relate these items in an adjacent block (Koufteros 1999).

Consequently, we conducted EFA utilizing both within and across-block designs to ensure the unidimensionality of the constructs. The factor structure was assessed using SPSS 22.0 software. We eliminated items based on the following criteria: items whose factor loading was less than 0.5, isolated items, and items which showed a high factor loading on several factors.

After assessing the factor structure of each theme individually, several items did not have adequate loadings and had significant cross-loading. Therefore, within block EFA eliminated 9 items, leaving 35 items for the final across block analysis. We conducted “Across block” EFA with all 35 items using maximum-likelihood extraction with varimax rotation. Consequently, 18 items were retained with 5 factors or constructs (see Table 3). We assessed the reliability analysis of the scale and its different dimensions by Cronbach’s alpha.

**Table 3:** Retained Items and constructs

Construct	Label	Communality Sample 1	Communality Sample 2
*Structure and mechanisms*			
Structure_1	Redesign of structures	.384	.625
Structure_2	Create adaptable structural design	.637	.756
Structure_3	Establishment of adaptive mechanisms transitions between the structural designs	.805	.818
Structure_4	Control of adaptive mechanisms transitions between the structural designs	.721	.781
*System development*			
System_1	Build sustainable operational systems	.455	.557
System_2	Development of systems able to respond to new market models (e.g., omnichannel)	.616	.766
System_3	Development of systems able to respond to new business models (e.g., circular economy)	.702	.755
System_4	Development of systems able to respond to positive disruptions (e.g., innovations)	.660	.718
*SC redesign*			
Redesign_1	SC Redesign should consider knowledge management	.486	.629
Redesign_2	SC Redesign can help to bypass problems in the SC	.507	.711
Redesign_3	SC Redesign should bring more visibility in the SC	.663	.719
*SC Feed back*			
Feedback_1	Implement a positive feedback cycle (= agility-oriented cycle)	.749	.755
Feedback_2	Implement a volatile feedback cycle (= resilience-oriented cycle)	.732	.818
Feedback_3	Implement a survivability feedback cycle (= survival-oriented cycle)	.457	.812
*SC Process*			
Process_1	Master / control basic SC processes	.629	.603
Process_2	Identify SC skills and training	.629	.603
Process_3	Implement S&OP process	.417	.664
Process_4	Set up KPIs	.449	.705

The results indicated that the items loaded most strongly onto their respective constructs. When the 18 items are integrated simultaneously into the factor analysis, they are projected on the five identified factors (factor loading > 0.5, no overlap between dimensions). Five factors representing SCV emerged from the analysis: Structure and Mechanisms, System Development, SC Redesign, SC Feedback, and SC Process, which mapped as follows on our original content and face validated constructs (cf. Table 2).

The final sample EFA explained 71.9% of the variance for a KMO of 0.875. In addition, the scale obtained a highly satisfactory alpha of 0.899. These results attest to the reliability of the overall scale of SCV. “Appendix 1” reports the means and standard deviations of each item. Criterion validity through Pearson correlations are presented in “Appendix 2”. Table 4 reports the results of the EFA. Univariate normality tests (not reported) showed limited skewness or kurtosis (Hair et al. 2010).

**Table 4:** Exploratory factor analysis

	Initial Sample N = 163					Final Sample N = 265				
*Items*	1	2	3	4	5	1	2	3	4	5
Structure_1	**.565**	.008	.006	.011	.211	**.752**	.118	.003	.007	.011
Structure_2	**.779**	.003	.010	.008	.111	**.854**	.002	.008	.021	.008
Structure_3	**.941**	.001	.009	.060	.010	**.898**	.011	.060	.010	.060
Structure_4	**.858**	.070	.003	.010	.004	**.849**	.002	.008	.004	.107
System_1	.019	**.612**	.005	.182	.201	.322	**.605**	.003	.020	.005
System_2	.118	**.766**	.010	.098	.039	.112	**.934**	.220	.018	.010
System_3	.009	**.872**	.009	.063	.069	.008	**.899**	.103	.015	.009
System_4	.010	**.710**	.061	.082	.073	.009	**.773**	.003	.030	.007
Redesign_1	.003	.010	**.586**	.060	.010	.028	.010	**.691**	.003	.009
Redesign_2	.020	.009	**.752**	.010	.004	.055	.009	**.890**	.220	.010
Redesign_3	.122	.160	**.751**	.042	.002	.111	.001	**.745**	.028	.004
Feedback_1	.004	.076	.088	**.710**	.221	.022	.122	.003	**.813**	.009
Feedback_2	.007	.052	.082	**.893**	.006	.102	.112	.220	**.917**	.019
Feedback_3	.039	.008	.004	**.856**	.087	.018	.068	.021	**.903**	.029
Process_1	.010	.002	.068	.001	**.701**	.005	.0333	.028	.118	**.699**
Process_2	.009	.003	.072	.006	**.598**	.009	.006	.059	.009	**.783**
Process_3	.010	.008	.068	.044	**.601**	.109	.022	.073	.018	**.677**
Process_4	.027	.220	.044	.039	**.658**	.087	.201	.009	.007	**.802**
Cronbach’s Alpha by factor*	.716	.799	.756	.896	.602	.853	.844	.772	.856	.690
Cronbach’s Alpha of the scale	.906					.899				
	KMO sampling adequacy = .868 Bartlett’s test of Sphericity χ^2^ = 2.813 df = 253 Sig = .000 Total variance extracted = 69.9% There are less than 3% non-redundant residuals in factor matrix with values greater than .05					KMO sampling adequacy = .875 Bartlett’s test of Sphericity χ^2^ = 2.952 df = 325 Sig = .000 Total variance extracted = 71.9% There are less than 3% non-redundant residuals in factor matrix with values greater than .05				

*In the case of scale development, Alpha’s values exceeding 0.6 are deemed acceptable (Dunn et al. 1994; Nunnally and Bernstein 1994)

#### Confirmatory factor analysis

After dropping the problematic items, we analyzed the remaining 18 items via CFA using Amos 24.0. In this case, each construct was linked to its associated item set from which the analysis was conducted. To assess the CFA results, we used three common fit indices: the comparative fit index (CFI), Tucker–Lewis index (TLI), and incremental fit index (IFI). The stated acceptability standard of 0.90 (Sharma 1996) was achieved. We used these because prior research has noted their stability across a wide range of data parameters (e.g., sample size) (Marsh et al. 1988; Hatcher 1994).

The results of this analysis indicated that the model fits the data well (χ^2^ = 236.22, df = 109, CFI = 0.942, TLI = 0.927, IFI = 0.942), which supports the conclusion that the individual constructs are unidimensional.

#### Reliability assessment

Reliability is the relative percent of variance in an observed variable that is accounted for by the true scores. However, the true score cannot actually be obtained, so a more accurate definition refers to the stability of the scores for a particular scale (Hatcher 1994). The most common reliability aspect is the assessment of internal consistency, where an internally consistent measure is the one with highly correlated items with both each other and the total scale (Hatcher 1994; Hair et al. 2010). The most common metrics used to assess internal consistency are Cronbach’s coefficient alpha and composite reliability (Hatcher 1994). In both cases, the expectation is that the individual construct score will exceed a value of 0.70 (Nunnally 1978). Average variance extracted (AVE) is one of the metrics used to assess the validity of constructs, but Malhotra and Dash (2011) argue that AVE is often too strict, and reliability can be established through CR alone.

We assessed each of the SCV constructs for both Cronbach’s alpha and composite reliability (Table 5). As is evident, all constructs exhibit acceptable levels in both metrics, which indicates that the newly created scales are reliable.

**Table 5:** Construct reliability AVE correlations and shared variance

Construct	(1)	(2)	(3)	(4)	(5)	Composite Reliability (CR)	Cronbach α	AVE
Structure and mechanisms (1)	**.782**	.284	.271	.214	.164	.858	.854	.606
System development (2)	.526	**.766**	.523	.314	.298	.850	.846	.579
SC redesign (3)	.442	.636	**.734**	.350	.402	.777	.775	.537
SC feedback (4)	.393	.428	.420	**.829**	.316	.868	.866	.682
SC Process (5)	.395	.532	.634	.559	**.770**	.762	.762	.591

Items in bold on the diagonal are the square root of AVE figures, the lower left triangle contains the construct correlations, and the upper right triangle contains the shared variance between constructs

#### Convergent validity assessment

Convergent validity is the extent to which varying approaches to construct measurement yield the same results (Campbell and Fiske 1959). In addition, it can refer to whether items comprising a scale behave as if they are measuring one common construct (Davis 1989). Convergent validity can be assessed using several different methods, with the most common ones including evaluating the multi-trait matrix or evaluating the measurement model for the constructs under consideration. As multiple methods were not used in this research, we used the CFA approach to assess convergent validity. Specifically, convergent validity is demonstrated when individual items load significantly on a single construct and the measurement model has acceptable fit statistics (Hatcher 1994). As demonstrated by the acceptable fit statistics of the measurement model (χ^2^ = 236.22, df = 109, CFI = 0.942, TLI = 0.927, IFI = 0.942) and the significant path loadings for all measured constructs, the measures demonstrate convergent validity (“Appendix 1”).

Beyond the model fit tests, convergence was also tested through the utilization of the AVE for each individual construct. In this case, the AVE should exceed a recommended threshold value of 0.50 to determine if the variance shared between the measurement items and the construct exceed the variance that would be explained by the individual measurement errors associated with each item. As is evident from Table 5, the scales meet the threshold, with the AVE values exceeding 0.50. Taken collectively, the tests undertaken provide assurance that convergent validity is supported.

#### Discriminant validity assessment

Scales demonstrate discriminant validity if the items of each construct only reflect that single construct (Bagozzi et al. 1991). Discriminant validity is the ability of a set of measurement items to differentiate between two related but conceptually different constructs. Discriminant validity is ensured when a scale does not measure the construct it was not intended to measure. Traditionally, a multi-trait matrix is used to assess discriminant validity (Campbell and Fiske 1959). However, more recent research has evolved to utilize factor analysis (Netemeyer et al. 2003) as well as the AVE test (Fornell and Larcker 1981).

We employed both EFA and CFA to provide initial support for the existence of discriminant validity. In this case, the EFA results establish the initial evidence for discriminant (and convergent) validity. The EFA is followed by the CFA, whereby the factor structure is verified via the utilization of a structural equation model (SEM) methodology. As noted above, the model fits the data well, which provides evidence that the scales are, in fact, discriminant. In addition, the AVE was calculated for each construct, and this value was compared with the shared variance between all the associated construct pairings. To demonstrate an appropriate level of validity, each individual AVE should exceed the squared correlation (shared variance) between constructs. The results (see Table 5) provide support for discriminant validity, as each AVE exceeds the squared correlation between construct pairs.

**Fig. 3:**
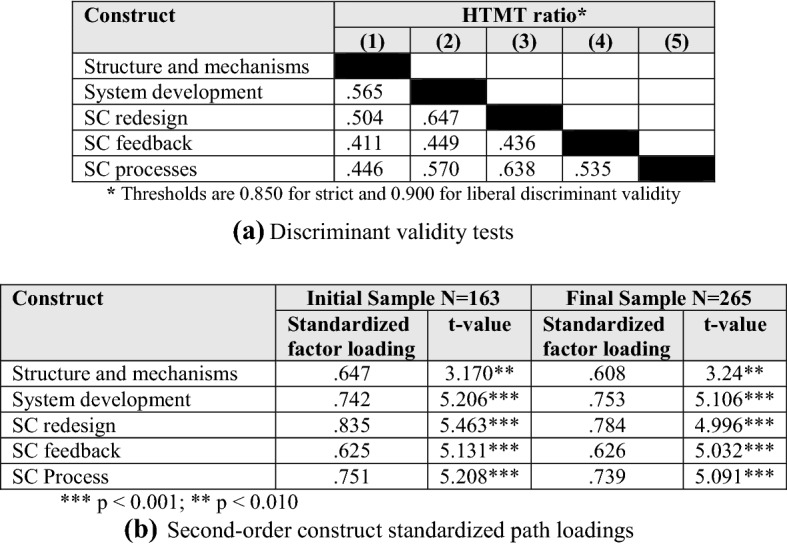
Discriminant validity tests and second-order construct standardized path loadings

We also tested discriminant validity employing the more current Heterotrait–monotrait (HTMT) discriminant validity analysis approach (Henseler et al. 2015). HTMT produces a ratio of the average correlations of indicators across two constructs relative to the geometric mean of the averages of the correlations of indicators within each construct. The analysis suggests that discriminant validity is achieved when the average item correlations within a construct are substantially greater than the average item correlations across constructs. An HTMT ratio < 0.85 suggests discriminant validity. HTMT has been shown to be a more reliable and powerful test of discriminant validity than traditional methods, such as the Fornell–Larcker criterion and the examination of cross-loadings (Henseler et al. 2015). The results presented in Fig. 3 confirm the discriminant validity of SCV constructs.

#### Second-order construct analysis

When theory suggests that the correlations among first-order constructs can potentially be more effectively explained by a higher-order factor, additional analyses can be conducted to test for the existence of a second-order construct. In the case of the SCV concept, there is no explicit guidance in the literature, as this is an emergent theoretical domain. Our conceptualization of SCV based on literature and earlier cited rationales, and subsequent operationalization, argues for SCV being a second order latent construct that is reflected in subordinate first-order constructs. An important note is that the higher-order factor is the theoretical explanation for the covariation of the first-order constructs (Segars and Grover 1999). Therefore, the second order model cannot exhibit an improved fit when compared to the correlated, first-order model. However, the low-level model can be used as the target fit for the high-level model, with the aim of providing a comparable fit via a more parsimonious, theoretically relevant model. The efficacy of this comparison can be examined through the utilization of a target coefficient (T), which is calculated as the chi-square of the first-order model divided by the chi-square of the second-order model [χ^2^ FirstOrder ÷ χ^2^ SecondOrder] (Marsh and Hocevar 1985). As the coefficient is a comparison of an “ideal” model to a competing model, it has an upper bound of 1.0, with higher numbers indicating that the relationship among the first-order factors is effectively being captured by the second-order model. Following the procedure of Segars and Grover (1999), each model’s chi-square value is adjusted for the degrees of freedom for the individual model (i.e., χ^2^/df). The adjusted χ^2^ for the first-order model is 2.17 (236.22/109), and the second-order model value is 2.27(259.097/114).

The target coefficient is then calculated to be 0.96, which lends support for the second-order model being a valid, parsimonious representation of the relationships between the first-order constructs. In addition, support for the second-order model is demonstrated by the paths between the first- and second-order constructs all being significant (see Fig. 3). According to the data of the final sample, SCV as a second order construct has a composite reliability of 0.831, an AVE of 0.50, and a maxR(h) of 0.834. Fit measures are also adequate:

**Table:** 

CMIN/DF	2.273	Between 1 and 3	Excellent
CFI	0.933	> 0.95	Acceptable
RMSEA	0.069	< 0.1	Acceptable

### Phase 4: Nomological validity assessment

The nomological validity (Churchill 1995) of the proposed scale of SCV was examined by testing the relationship with a related outcome construct: SC performance. Our nomological rationales are underpinned by the resource based view (RBV) and the contingent resource based view in particular (CRBV—Brush and Artz 1999). RBV is a theoretical approach that emerged as a response to the turbulence in the business environment, accentuated by crises (Wernerfelt 1984; Barney 1991, 2012; Peteraf 1993). RBV attributes firm specific competitive advantage to the possession and deployment of scarce, valuable and inimitable resources. Despite its popularity, RBV has been criticized by some scholars for the ambiguity of the resources’ concept, its static approach (Priem and Butler 2001a, b), difficulty to be operationalized (Bromiley and Rau 2016), and context insensitivity (Ling-Yee 2007; Brandon-Jones et al. 2014). On the other hand, the CRBV integrates the idea of a dynamic environment and suggests that a competitive advantage may be contingent: some specific conditions have a significant effect on the impact of resource bundling and capability building (Brandon-Jones et al. 2014). In the SCM research field, the contingent perspectives of RBV enables to consider the necessity to adapt firm’s resources and capabilities to the environment in order to achieve a better SC performance (Brandon-Jones et al. 2014; Eckstein et al. 2015; Dubey et al. 2020). Additionally, the way SC resources and capabilities could be bundled in order to align with external contingency such as a dynamic environment punctuated by severe disruptions and uncertainties (Aragon-Correa and Sharma 2003) is still an understudied research area (Brandon-Jones et al. 2014).

Our psychometric treatment of SCV identifies five specific resources and capabilities, viz. Structure and Mechanisms, System Development, SC Redesign, SC Feedback, and SC Process that firms could develop and dynamically orchestrate to improve survivability over time during acute disruptions (Eddleston et al. 2008; Blackhurst et al. 2011; Queiroz et al. 2020). To wit, from the RBV perspective, such resources and capabilities can be considered as valuable, rare, inimitable (these are in the main high-level organizational capabilities and difficult for competitors to mimic or replicate at least in the short to medium run—Barney 1991), and dynamic (Dubey et al. 2021). Moreover, by mobilizing the CRBV (Brush and Artz 1999), these five specific resources and capabilities can be combined in such a way as to be able to respond to external contingencies, i.e., a particularly uncertain environment.

RBV posits that firms can achieve sustained competitive advantage with the possession and use of valuable, inimitable, and non-substitutable resources (Hart 1995). Additionally, based on the literature, we argue that the SCV resources and capabilities possessed by the firm may have impact on SC performance under the contingency of severe disruptions. Thus, we theorize a positive relationship between SCV and SC performance (Ivanov 2020b; Ivanov and Dolgui 2020b).

SC performance is measured using the following items: order fulfilment, delivery as promised, delivery flexibility, flexibility to change output volume (Chae et al. 2014), all reported relative to competition. The selection of this scale is based on two factors. First, these performance indicators reflect the adaptation abilities of an SC as manifested in SCV. Second, this scale has been cited extensively in past research.

**Fig. 4:**
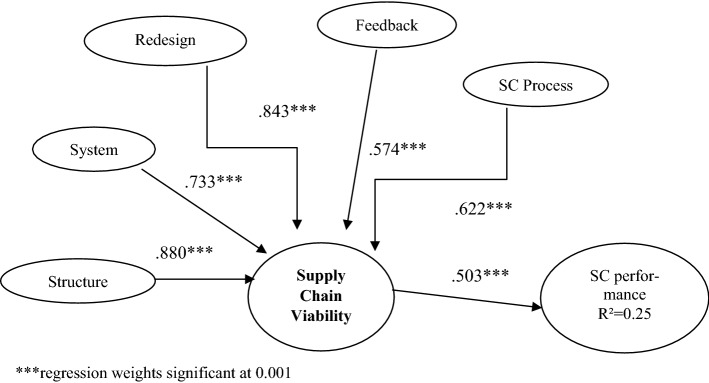
Research model for nomological validity

We collected data using a third and independent sample of 119 respondents to conduct the nomological validity analysis of the proposed SCV measurement scale. Figure 4 exhibits the model and the path coefficients.

The fit indexes of the proposed model are acceptable with χ2 = 441.61, Degrees of freedom (*df*) = 309, *p* < 0.001, CFI = 0.901, TLI = 0.854, IFI = 0.873, RMSEA = 0.06 (see Table 6).

**Table 6:** Fit indexes for nomological validity

Fit statistics	Statistics	Recommended range*
χ^2^/*df*	1.429	< 3.0
RMSEA	.06	< 0.1
PGFI	.705	> 0.5
PNFI	.772	> 0.5
TLI	.854	> 0.9
CFI	.901	> 0.9

*References: Azadegan et al. (2020), Hair et al. (2010)

As theorized, SCV is significantly and positively related to SC performance (0.503 path coeff—see Fig. 4), providing support for the nomological validity of our SCV scale. Collecting the preceding discussions, we describe a defined, systemic phase driven scale development process, commencing with construct operationalization and instrument generation, and concluding with an examination and verification of scale psychometric properties. We emerge with a scale that measures the concept and construct of SCV with substantive content validity, scale reliability, and convergent, discriminant and nomological validity. Our sampling plan imparts diversity and rigor to the scale. We triangulated data collection to include interviews, field visits, and separate, repeated surveys. Our data is cross-sectional in industry scope, and sourced from business respondents with considerable experience and expertise in the SC and operations functions, lending added authenticity as well as external validity to our scale.

## Discussion of results and implications

### Implications for theory

Our findings make several contributions to the SC literature. First, we add empirical tangibility to the conceptual notion of SCV, building on, and extending theory. We introduce a rigorously developed and diligently validated scale for measuring SCV. Future studies that extend research on this nascent construct should find our scale to be of use. Second, we develop a hierarchical model of SCV. The validated SCV measurement scale is a second order construct which contains five unidimensional constructs, namely “structures and mechanisms,” “systems development,” “SC redesign,” “SC feedback,” and “SC processes.” The scale adds to the body of knowledge by identifying key resources and dynamic processes required for fostering SCV. Further, the SC orientation of the scale is consistent with, and reinforces the general belief that RBV should not be limited within internal organizational boundaries (Paulraj 2011; Hitt et al. 2016). Finally, this scale is a way of bundling the SC resources and capabilities in the face of external contingencies, in this case "super disruptions" such as those generated by the COVID-19 pandemic. Thus, this study contributes to the stream of SCM research that seeks to demonstrate the usefulness of CRBV as a theoretical perspective (Grötsch et al. 2013; Brandon-Jones et al. 2014; Dubey et al. 2020).

We dropped several items during scale content validation and psychometric evaluation. This has implications for theory. SCV research is emergent (Ivanov 2018b, 2020b; Ivanov and Dolgui 2020b) and thus, just as with any exploratory topic, there are, and will be, conflicting concepts which may in turn bring some redundancies in pre-existing concepts. Building a measurement scale enables us to consider, specify, and examine key elements of a theoretical concept. However, this is a dynamic process and we fully anticipate that our scale will see changes as contingencies are applied in the future. Some of the items dropped were of interest to the interviewees, but did not survive statistical analysis. It is conceivable that such items may re-emerge in other contexts or may be linked to SCV in some other, still unexamined, way. Future research may look into such possibilities.

A consequential finding is the positive and significant nomological link between SCV and SC performance. Admittedly not the central focus of this research, this relationship harbors important meaning for theory and practice—and offer interesting opportunities for future research. Another distinguishing feature of our study is that data collection was confined to business respondents with maturity and experience in the SC and operations functions. We did not seek or collect data from students or other proxies. The scale is thus built on real world business data foundations, and will therefore reflect reality and relevance to future respondents and researchers.

### Implications for practice

An important finding from a practitioner perspective is the positive and significant link between SCV and SC performance. SCV builds a set of capabilities that enable quick pivots and adaptation in response to super-disruptions. These capabilities, by virtue of being at the organizational level, are rare, valuable and difficult to imitate—and can thus create firm specific competitive advantage, if exploited with appropriate strategies. Global super disruptions such as the COVID-19 outbreak demand that SC professionals look beyond SC stability, robustness, resilience, agility, flexibility, or even efficiency. Resources should be developed and/or acquired and processes should be setup to build SCV. SC professionals may find our validated SCV measurement scale useful in this regard—initially perhaps as a diagnostic tool, to identify areas that require specific improvements. More precisely, the SCV measurement scale shows the considerable need to: (1) focus on key SC resources and processes (2) to adapt the SC design by using feedback cycles in order to face environmental changes and uncertainties. Besides, undergoing the steps involved in the process of evaluating or developing SCV would in itself provide a useful mechanism for preparedness, collaboration and awareness of risks and opportunities in the supply base, internal processes and market and ecological systems. The next SC trauma could be just around the corner.

## Conclusion

The concept of SCV with its emphasis on adaptation and survival has attracted attention from COVID shocked industry. However, the relative novelty of the concept makes defining the pathway to reach SCV a difficult task. Our study provides definition to this endeavor. We define SCV and associated dimensions, and develop new multi-item measurement scales for measuring these constructs. Unlike prior, our study is purely empirical, obtaining and analyzing SC professionals’ perspectives on SCV. A secondary contribution of this work is the demonstration of a rigorous empirical scale and item development process. Our validation of a measurement scale for SCV also provides a degree of clarity on the differences between the SCV and overlapping concepts like SC resilience.

As in most research, this study has some limitations. The use of a convenience sample for interviews during the qualitative content and face validation phase may have limited our insights early in the research process. The use of a convenience sample at this first step is justified by the well-known disadvantage of conducting scale-development research, namely the time commitment required (Hensley 1999). However, we used random sampling for the three subsequent quantitative data collections, alleviating concerns regarding this issue. Our scale is certainly not final in any way—future studies will, we hope, test, refine, and improve the scale in different conditions and contexts. Nonetheless, we believe that the rigor of our scale development process makes the SCV foundational dimensions contingent-agnostic to an extent.

Despite these limitations, we believe our SCV scale will provide researchers with a robust construct measurement mechanism scales when investigating this emerging concept (Ivanov 2018b, 2020). Industry would also benefit from considering these measurements scales as diagnostic tools and pathways in designing viability into SCs. In addition, we believe that both industry and academia are likely to benefit from new research on how SCV is related to other concepts such as SC digitalization (Blackhurst et al. 2021; Ivanov et al. 2020a, b; Zouari et al. 2021). To do so, the new clearly defined constructs and robust measurement scales of SCV will assist these future investigations. Finally, some “classic” SCM fundamentals feature in the SCV measurement scale. More research is required to fully understand how and why such SCM fundamentals integrate into the concept of SCV. For example, in the area of SC design, the cost-efficiency oriented models can be extended by multi-objective functions and multi-level viable SC designs. We consider these issues to be future research opportunities. We expect that our study and operationalization of SCV will stimulate further theory development as researchers begin developing SCV centered nomological frameworks, and use the SCV scale to test such conceptualizations. Research on possible negative aspects of SCV, SCV development and maintenance expense, SCV sustainability, and the SCV development process (particularly when visibility and control is often limited to a Tier 1 or Tier 2 supply level) would be of interest and utility.

## Appendix 1: Sample analysis

**Table:**

Construct	Sample 1 N = 163		Sample 2 N = 265	
	Mean	S.D	Mean	S.D
EFA retained items means and standard deviations				
*Structure and mechanisms*				
Structure_1	5.48	1.157	5.25	1.2020
Structure_2	5.07	1.311	5.68	1.147
Structure_3	5.74	1.098	5.26	1.208
Structure_4	5.66	1.073	5.02	1.114
*System development*				
System_1	5.71	1.286	5.88	1.278
System_2	5.61	1.167	5.92	1.174
System_3	5.54	1.228	5.81	1.229
System_4	5.79	0.954	5.98	1.108
*SC redesign*				
Redesign_1	5.66	1.182	5.59	1.385
Redesign_2	5.34	1.292	5.50	1.303
Redesign_3	5.63	1.160	5.67	1.229
*SC Feed back*				
Feedback_1	5.75	1.096	5.86	1.088
Feedback_2	5.66	1.073	5.70	1.233
Feedback_3	5.66	1.073	5.72	1.275
*SC Process*				
Process_1	5.58	1.257	6.36	0.920
Process_2	6.06	0.914	6.18	1.126
Process_3	6.12	1.108	6.55	0.900
Process_4	6.11	1.107	6.50	0.887

Construct	Sample 1		Sample 2	
	Standardized path loading		Standardized path loading	
Nomological analysis: Item loadings				
*Structure and mechanisms*				
Structure_1	0.549		0.527	
Structure_2	0.775		0.736	
Structure_3	0.927		0.916	
Structure_4	0.889		0.887	
*System development*				
System_1	0.668		0.673	
System_2	0.821		0.797	
System_3	0.814		0.793	
System_4	0.806		0.795	
*SC redesign*				
Redesign_1	0.727		0.702	
Redesign_2	0.692		0.681	
Redesign_3	0.769		0.813	
*SC feedback*				
Feedback_1	0.798		0.788	
Feedback_2	0.877		0.877	
Feedback_3	0.847		0.821	
*SC processes*				
Process_1	0.705		0.584	
Process_2	0.789		0.633	
Process_3	0.675		0.753	
Process_4	0.609		0.687	

## Appendix 2: Pearson Correlation tests of combined-sample inter-items (N = 163 + 265 = 428)

**Table:**

	St1	St2	St2	St4	Sy1	Sy2	Sy3	Sy4	Re1	Re2	Re3	Fe1	Fe2	Fe3	P1	P2	P3	P4
Structure_1	1																	
Structure_2	.574**	1																
Structure_3	.440**	.673**	1															
Structure_4	.445**	.610**	.825**	1														
System_1	.263**	.391**	.405**	.395**	1													
System_2	.202**	.371**	.346**	.360**	.546**	1												
System_3	.232**	.342**	.342**	.316**	.509**	.664**	1											
System_4	.232**	.400**	.372**	.338**	.493**	.622**	.643**	1										
Redesign_1	.223**	.285**	.287**	.310**	.330**	.328**	.342**	.478**	1									
Redesign_2	.285**	.269**	.226**	.263**	.320**	.295**	.284**	.373**	.474**	1								
Redesign_3	.320**	.352**	.284**	.301**	.380**	.403**	.342**	.442**	.552**	.578**	1							
Feedback_1	.256**	.297**	.305**	.331**	.277**	.231**	.294**	.378**	.348**	.315**	.276**	1						
Feedback_2	.158*	.268**	.244**	.281**	.254**	.208**	.258**	.281**	.267**	.256**	.292**	.691**	1					
Feedback_3	.171**	.243**	.333**	.314**	.285**	.260**	.315**	.350**	.249**	.157*	.212**	.627**	.731**	1				
Process_1	.209**	.188**	.210**	.195**	.271**	.221**	.272**	.287**	.290**	.278**	.298**	.381**	.333**	.286**	1			
Process_2	.307**	.345**	.280**	.262**	.308**	.254**	.241**	.260**	.371**	.318**	.343**	.479**	.430**	.420**	.570**	1		
Process_3	.239**	.240**	.233**	.193**	.272**	.283**	.345**	.374**	.283**	.244**	.355**	.234**	.180**	.158**	.406**	.359**	1	
Process_4	.178**	.205**	.166**	.214**	.356**	.319**	.255**	.313**	.273**	.240**	.442**	.255**	.216**	.168**	.387**	.447**	.498**	1

*Correlation significant at *P* < .05

**Correlation significant at *P* < .01

## Footnotes

1 Commonalities and differences between SC resilience and SCV can be illustrated in the following simple format. In an operational view, we are driving cars to get faster at some destinations. Strategically, we are using cars to stay mobile. If a car breaks down then it should be repaired. For the time of service, one can receive another car as a substitute. Then we receive the car back in an old, normal (or equivalent) state. This is a classical resilience profile “disruption—backup—recovery—old normal”. However, if the car cannot be repaired any more, we should adapt structurally. One can start using public transportation to stay mobile. Or one can purchase a new car. In both cases, the decisions to adapt are driven by the strategic objective to stay mobile rather than by the operational objective to get faster at some destination. And this is the viability profile: “disruption–performance and structure degradation—adaptation-to-survive (i.e. to ensure/secure some strategically important service; the mobility)—search for “new normal”—stabilization in the “new normal”—performance recovery”.
